# The health risks of dysphagia for patients with head and neck cancer: a multicentre prospective observational study

**DOI:** 10.1186/s12967-021-03144-2

**Published:** 2021-11-22

**Authors:** Maria Giulia Cristofaro, Ida Barca, Francesco Ferragina, Daniela Novembre, Yvelise Ferro, Roberta Pujia, Tiziana Montalcini

**Affiliations:** 1 Department of Experimental and Clinical Medicine, Maxillofacial Surgery Unit, “Magna Graecia” University, Viale Europa, 88100 Catanzaro, Italy; 2 Department of Health Science, Nutrition Unit, “Magna Graecia” University, Viale Europa, 88100 Catanzaro, Italy

**Keywords:** Maxillofacial surgery, Malnutrition, Enteral nutrition, Dysphagia, Head and neck cancer

## Abstract

It is well known that malnutrition is a frequent co-morbidity in cancer patients, especially in those with head and neck neoplasms. This may be due both to the presence of dysphagia symptoms and to the appearance of adverse effects on chemotherapy and / or radiotherapy. The aim of this retrospective observational multicentric study is to evaluate the nutritional status between dysphagia cancer patients and non-dysphagia cancer patients. Data from 60 patients were analysed, 31 of which without dysphagia and 29 with dysphagia. Results highlight that patients with dysphagia had higher involuntary body weight loss than non-dysphagia ones (p < 0.001). By analysing the entire population, it stands out a weight loss rate of 12 ± 9% compared to the usual weight was observed and a prevalence of moderate / severe malnutrition diagnosis of 53%. Furthermore, 76% of the population who manifested the symptom of dysphagia presented severe malnutrition already at the first visit, compared to 32% of non-dysphagia subjects.

## Background

Malnutrition is one of the most frequent comorbidities in the patient with head and neck cancer. This altered nutritional status can be induced by cachexia, which is often associated with malignant tumours [1], dysphagia and/or odynophagia [2, 3], the iatrogenic effect of the treatment used (surgery, chemotherapy and/or radiotherapy) and harmful social behaviours, such as heavy smoking, poor diet and alcohol abuse, which are often associated with this patient group [4–6]. Dysphagia is a disorder of swallowing solid, liquid or semi-liquid foods; it is often associated with a dysfunction of the digestive system with incorrect transit of the bolus in the upper digestive tract. It may be associated with persistent hoarseness and dysphonia, pain, swelling in the neck, nosebleeds with respiratory obstruction or headache, chronic sinusitis unresponsive to treatment with antibiotics, numbness, or paralysis of the facial muscles. Dysphagia represents a weighty health problem as it makes it difficult to implement both an independent and safe oral diet.

Malignancies are usually treated through surgery, chemotherapy, and radiotherapy, which can be given individually or, more commonly, in combination. All these methods can increase the incidence of diet-related problems [7–9]. Moreover, the use of chemotherapy or radiotherapy, especially when combined, can increase the incidence of adverse effects, such as oropharyngeal mucositis, odynophagia, taste disturbances, xerostomia, nausea, vomiting and fatigue; all of these can contribute to dehydration and significant weight loss [10, 11], impaired nutritional status, functional capacity, and quality of life [12, 13]. Dysphagia is certainly the most frequent sequela of head and neck cancer (HNC) and its treatment. Oropharyngeal dysphagia is present in approximately 50% of patients with HNC; patients with total glossectomy and chemoradiotherapy has the highest rate of dysphagia. Also, malnutrition is present in about 20–50% of patients [14]. Indeed, weight loss has been shown to be a predictor of survival, with malnutrition associated with a poor prognosis for patients with head and neck cancer [15, 16]. The risk of malnutrition in cancer patients of maxillofacial surgery is therefore high, and support with enteral nutrition, now considered the standard of care [17], becomes essential. Both the nasogastric tube (NG) and the percutaneous endoscopic gastrostomy tube (PEG) are equally effective in providing the fluids and nutrients needed by patients with head and neck cancer undergoing treatment [18, 19].

Therefore, the aim of the study was to evaluate the nutritional status, in particular the prevalence of malnutrition, between dysphagia cancer patients and non-dysphagia cancer patients.

## Methods

A total of 60 patients (39 men and 21 women) agreed to take part in this prospective observational multicentric study: patients from the three Hub Hospitals (afferent even from the various spoke hospitals in the area) and the only University Hospital in the Calabria region were evaluated and sent to the Maxillofacial Unit of University “Magna Graecia” of Catanzaro. They were all evaluated in collaboration with the Clinical Nutrition Unit, between January 2020 and March 2021. This study was conducted according to the guidelines set out in the Declaration of Helsinki.

### Inclusion and exclusion criteria

The study inclusion criteria included patients of both sexes, aged > 18 years and diagnosed with oral, laryngeal, and pharyngeal cancers. Instead, who had not completed the diagnostic process or with secondary disorders, were excluded.

### Search strategy

Each patient underwent both a maxillofacial surgical and a nutritional examination. During the maxillofacial visit, a thorough medical history was first carried out, investigating the familiarity, risk factors and therapies taken by the patients; this was essential for patients who already presented dysphagia at the time of diagnosis. In fact, it is well known that dysphagia can also be secondary to polypharmacy. The pre-surgical procedure continued with the prescription of first level (such as ultrasound of the neck and salivary lodges), second level (MRI and CT of the facial massif and neck with and without contrast medium), and third level (PET-TC total body) imaging examinations. During the nutritional visit, patients were subject to the following nutritional status assessments: (1) measurements of anthropometric parameters such as weight, height, BMI, arm circumference and triceps fold; (2) muscle function evaluation such as strength of handshake, calculated through the hand grip test; (3) body composition analysis such as the amount of total body water (TBW), fatty free mass (FFM), fat mass (FM), skeletal muscle mass (SMM), skeletal muscle mass indicated for height (SMI) and skeletal appendicular muscle mass (ASMM). Patient Generated Subjective Global Assessment (PG-SGA) screening test was made, and patients were considered mildly malnourished for 5–10% weight loss, moderately malnourished for 10–20% weight loss and severely malnourished for > 20%. Finally, the patients underwent bioimpedance analysis (BIA 101 RJL/Akern; Detroit, USA/Florence, Italy) which assessed the resistance, reactance, and phase angle; the instrument then provided, through predictive equations provided by the manufacturer using the BodyGram Pro 3.0 software (Akern), data relating to body composition. Thanks to these parameters the diagnosis of sarcopenia was made. The following blood chemistry tests were recorded for each patient: glycemia, creatinine, HDL cholesterol, triglycerides, total proteins, albumin, calcium, sodium, phosphorus, iron, globules white, red blood cells and haemoglobin. All patients were re-evaluated one year later. Moreover, all patients underwent an anti-inflammatory balanced diet with whole grains high in fibres, polyphenol-rich vegetables, and omega-3 fatty acid-rich foods.

### Trial procedures

The study was approved by the Ethics Committee of Magna Graecia University of Catanzaro (Reference number 302 of 17 October 2019). All patients signed informed consent to participate in the study. An investigator amply clarified the details of the study and explained that participation in the study was voluntary and that individuals could not be identified. Furthermore, potential parties were given ample opportunity to ask questions. Participation in the study was 100%.

### Statistical analysis

Descriptive data were calculated as frequencies (%) and are presented as means with standard deviations for normally distributed variables. To detect a difference in the prevalence of malnutrition of at least 40% at the first visit between the patients with dysphagia compared to non-dysphagia subjects, with a power of 80% and a two-sided significance level of 0.05, a minimum of 22 participants each group were required. Student's t-test for independent samples was used to evaluate significant differences in population means divided according to the diagnosis of dysphagia. The chi-square test was performed to compare the prevalence in the population classified according to the presence of dysphagia. Statistical significance was accepted at p < 0.05. Where percentages do not add up to 100, this is because data have been rounded to the nearest 1%. Data in the text, tables and figures are presented as means with standard deviations. The analysis was performed using the statistical software SPSS 20.0 for Windows (S. Wacker Drive, Chicago, Illinois 60606, USA).

## Results

Data from 60 patients with a diagnosis of head and neck cancers were analysed for the study. The patients had a mean age of 69 ± 12 years and 65% of the population analysed was male. The mean duration of the disease before diagnosis was 153 days (27 days–1 year). In relation to the tumour site, 50% (n = 30) had tumours of the oral cavity, 35% (n = 21) of the pharynx and 15% (n = 9) of the larynx. Solid and liquid dysphagia was present in 48% (n = 29) of subjects. They all underwent surgery, only 56.67% (n = 34) then underwent chemotherapy / radiotherapy treatment. In the whole study population, there was a percentage of lost weight compared to the usual weight of 12 ± 9% and a prevalence of the diagnosis of moderate/severe malnutrition of 53%. The patients were divided into two groups: a group without dysphagia (51.67%, n = 31) and B group with dysphagia (48.33%, n = 29). Of group B, 18 patients (62.07%) had already presented for our observation with dysphagia symptoms; 11 patients (37.95%), on the other hand, developed dysphagia at a later time. Data relating to the general characteristics of the population divided into the two groups are shown in Table 1.

There was a higher prevalence of males in group B than in group A (p = 0.032). Besides patients with dysphagia (group B) had higher involuntary body weight loss than non-dysphagia patients (group A) with p < 0.001; they also had a lower BMI, arm circumference, and triceps bend value. Regarding body composition, patients in group B had a higher percentage of body water (62.3% compared to 55.4% of Group A) and a lower percentage of fat mass (16.4% compared to 25.7% of Group A) than group A (Table 1). Table 2 shows the data relating to the blood chemistry parameters.

**Fig. 1:**
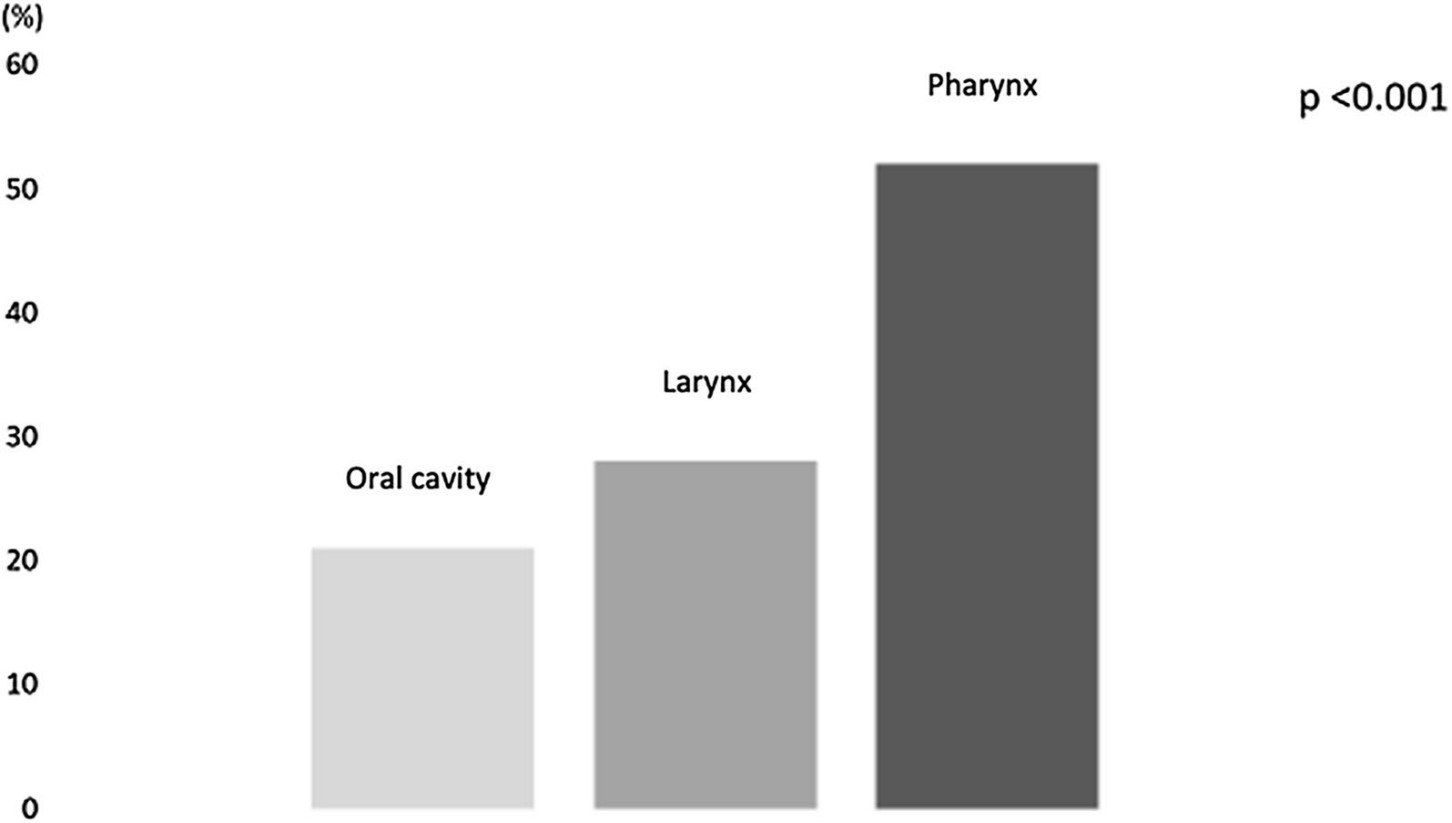
Prevalence of the diagnosis of dysphagia based on the anatomical location of the tumour

In the population analysed we found a higher prevalence of dysphagia in subjects with pharyngeal cancer (52%) compared to patients with cancer localized in the larynx (28%) and oral cavity (21%) (p < 0.001) (Fig. 1).

**Fig. 2:**
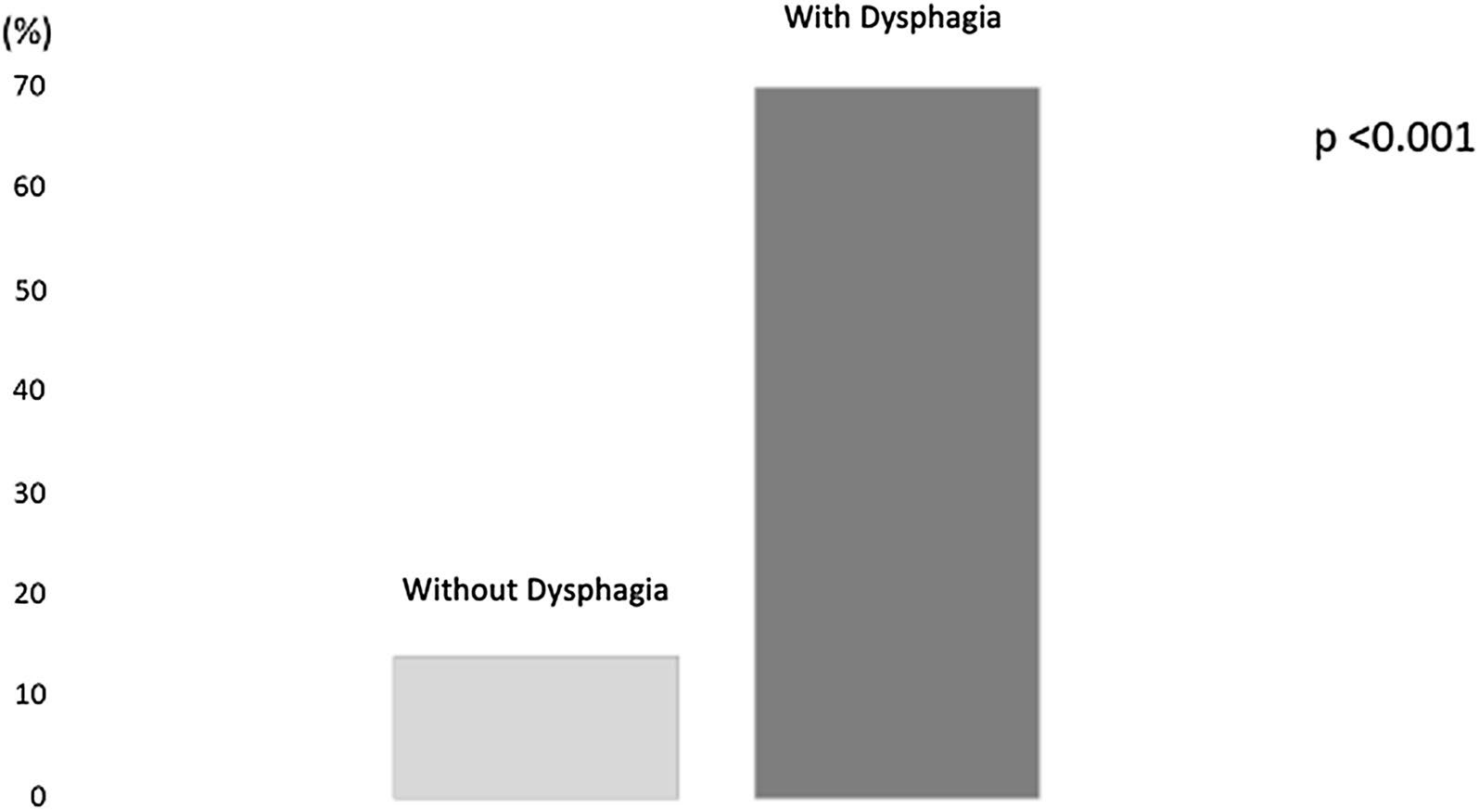
Prevalence of chemotherapy and/or radiotherapy in the population divided according to the diagnosis of dysphagia

In the population analysed there is a higher prevalence of chemotherapy and / or radiotherapy treatment in patients with dysphagia compared to non-dysphagia subjects (70% vs 14%, respectively; p < 0.001) (Fig. 2).

**Fig. 3:**
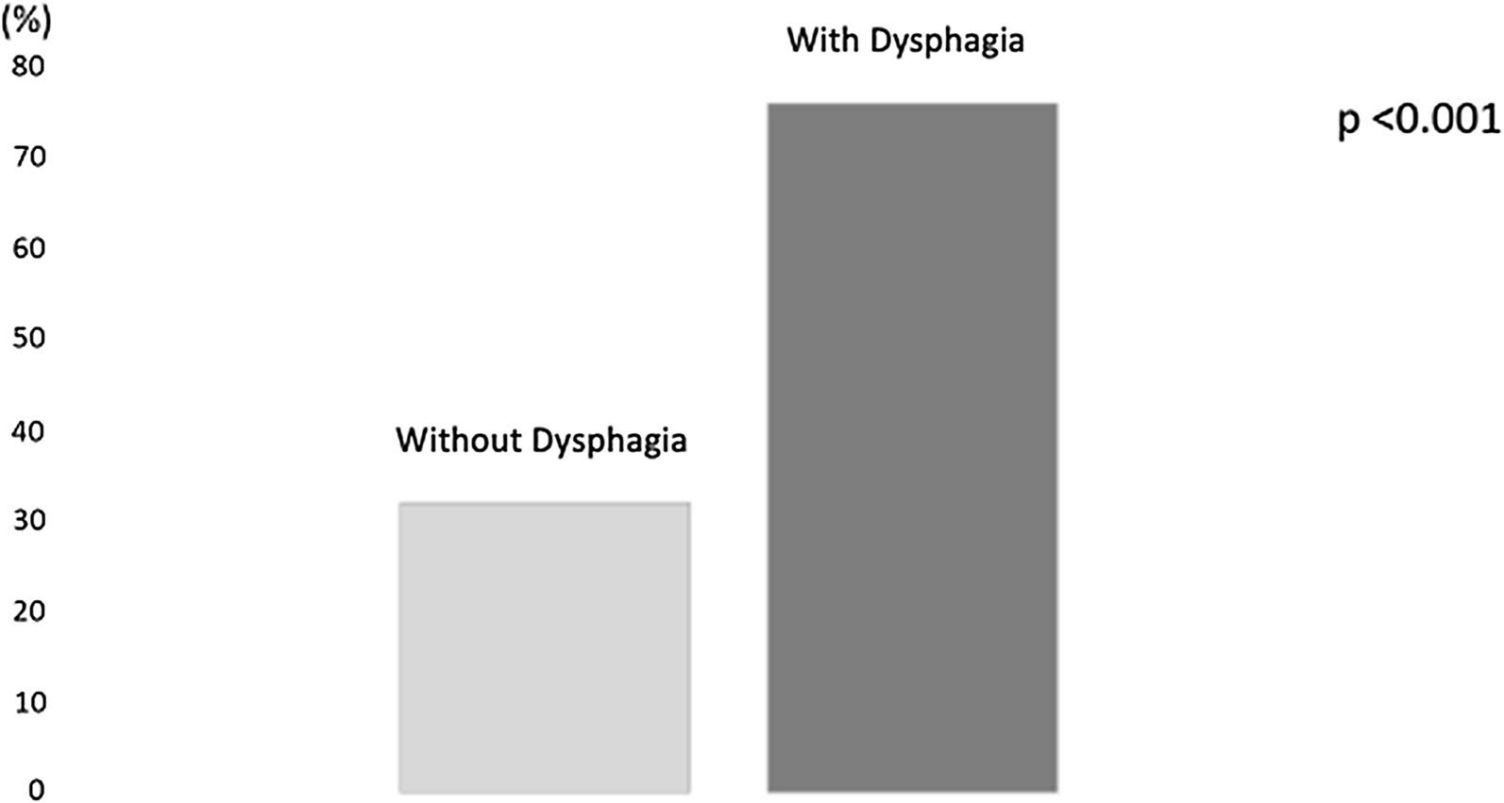
Prevalence of severe malnutrition in patients with- and without dysphagia

Figure 3 shows the prevalence of severe malnutrition in the population divided into the two groups: 76% of the dysphagia population (group B) had severe malnutrition already at the first visit, compared to 32% of the non-dysphagic subjects (group A) (p < 0.001).

**Fig. 4:**
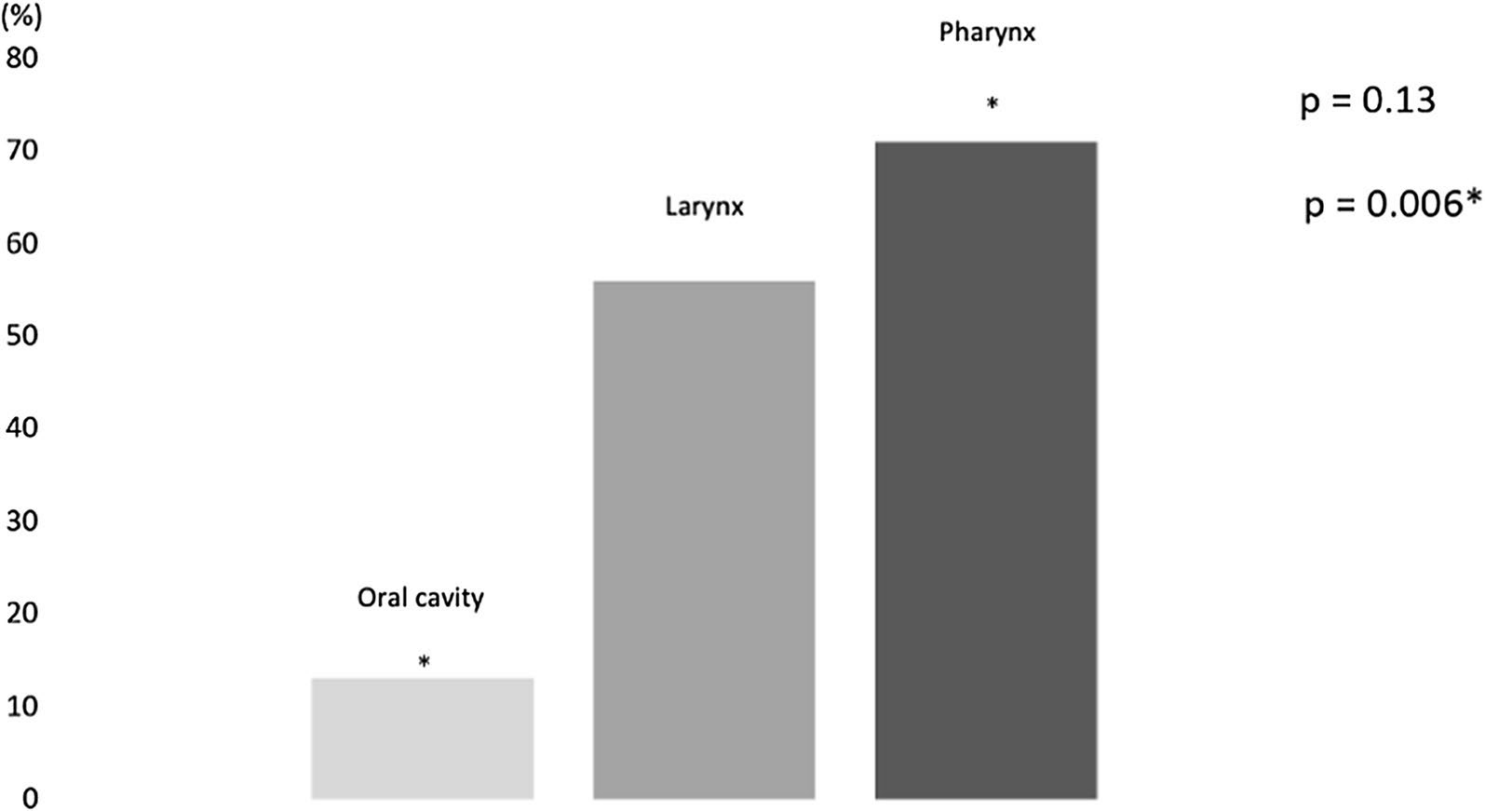
Prevalence of moderate/severe malnutrition based on anatomical location of the tumour

Moderate/severe malnutrition was 13% in patients with oral cancers, 56% of the larynx and 71% in those with pharyngeal cancers, which was not significant among the three groups (p = 0.13) but is differ significantly between pharyngeal and oral cavity cancers (p = 0.006) (Fig. 4).

Finally, in the population analysed, there was no statistically significant difference in the diagnosis of protein malnutrition between subjects with and without dysphagia (27% vs 12%, respectively; p = 0.29), nor in the diagnosis of sarcopenia between subjects with and without dysphagia (61% vs 50%, respectively; p = 0.73). All patients were re-evaluated one year later, and it was finally noted that: as regards group A, six patients (19.35%) had died, ten patients (32.26%) had severe malnutrition and fifteen patients (48.39%) had moderate malnutrition; as regards group B, seventeen patients (58,62%) had died and twelve patients (41.38%) had severe malnutrition.

## Discussion

In cancer patients, weight loss is the consequence of different pathophysiological processes that can coexist in the same patient. Precisely, in patients with HNC there is a reduction in nutrition both directly (mechanical obstacle for the digestive tract) and indirectly (inhibiting substances that act on the peripheral hypothalamic receptors). The appearance of side effects (pain, mucositis, dryness, fibrosis, important change in taste and appetite, etc.) following the treatments of chemotherapy and / or radiotherapy also plays an important role. [19–21]. Specifically, we have seen that in the examined sample (sixty patients), 13 (21.67%) already presented inappetence at the time of the nutritional visit. Instead, 5 patients (8.33%) presented with odynophagia at the time of the nutritional visit. Therefore, in 21.67% of cases the patient did not present the urge to eat; in 8.33% of cases the patients had a desire to eat but pain prevented its implementation. The prevalence of malnutrition in patients with head and neck cancer is approximately 20–50% [14, 22]. Regardless of the underlying mechanisms, weight loss and cancer-related malnutrition are multidimensional manifestations that reduce patient well-being, tolerance, and prognosis after antineoplastic therapy, decrease immunological responses to cancer cells and resistance to infections, and increase susceptibility to postoperative complications. disability and overall cost of care [23]. Also, swallowing problems resulting from radiotherapy can lead the patient to a state of chronic malnutrition, long-term need for enteral feeding (PEG), bolus inhalation with increased risk of aspiration pneumonia [24].

Patients, after having undergone surgery with any chemotherapy and/or radiotherapy, were fed on the second post-operative day by means of a nasogastric tube (SNG). The advantages of enteral nutrition on total parenteral nutrition are now well established: from a pathophysiological point of view, the anatomic-functional integrity of the intestinal mucosa is maintained, there is a better use of nutritional substrates, it is easier and safer and has less cost [25]. It is in fact necessary to guarantee the safety of the patient, avoiding the passage of food in the respiratory tract, to provide adequate nutritional quantities to prevent or correct the state of malnutrition and / or dehydration.

Immuno-nutrition was used in all 60 patients. It aims to strengthen the patient's immune defences, so as to reduce post-operative complications, in particular infections. We are therefore talking about artificial nutrition based on solutions enriched with nutrients with the aim of stimulating the host's immune response, improving the control of the inflammatory response and increasing nitrogen balance and protein synthesis after major surgery [26, 27]. The immune-nutrients used are glutamine, arginine, polyunsaturated fatty acids (omega-3), nucleotides, taurine, vitamins A, E and C, beta-carotene and trace elements (zinc and selenium).

In this multicentre retrospective observational study, 60 patients diagnosed with head and neck cancer, namely cancer of the oral cavity, larynx, and pharynx, were analysed. These patients were then divided into two groups, a Group A comprising patients without dysphagia and a Group B comprising patients with dysphagia. All of them underwent a surgery to remove the neoplasm. In line with the guidelines and according to the degree and stage of the disease, a chemotherapy and/or radiotherapy treatment was also proposed. Furthermore, they all underwent a thorough pre- and post-intervention nutritional examination. The first evaluation of the nutritional status highlights that in 53% of subjects with cancer of the oral cavity, larynx and pharynx, a diagnosis of moderate / severe malnutrition was made, with an average body weight loss of 12%. 48% of the subjects analysed had a diagnosis of dysphagia due to solids and liquids; the prevalence of dysphagia found in the study is like that found in other studies [14, 28]. The study shows that patients diagnosed with head and neck cancer and with the simultaneous presence of dysphagia, already suffered moderate malnutrition (weight loss of 16% vs usual weight), therefore with a significant weight loss bodily, when diagnosis was formulated. In particular, it was found that as many as 76% of dysphagia subjects had a diagnosis of moderate/severe malnutrition, compared to 32% of non-dysphagia subjects. Also, other nutritional indices suggest the presence of malnutrition such as reduced values of albumin, white and red blood cells, and haemoglobin. The evaluation of this population shows a higher prevalence of dysphagia in patients with pharyngeal cancer (52%) compared to patients with cancer localized in the larynx (28%) and oral cavity (21%). Furthermore, 70% of patients with dysphagia underwent chemo-radiotherapy treatments compared to 14% of non-dysphagia subjects.

## Conclusion

Malnutrition and dysphagia are very common symptoms in patients with HNC. In these patients, malnutrition is still an underdiagnosed and undertreated clinical condition. These patients, in fact, come to clinical evaluation when a state of moderate or severe malnutrition is already present and often when the symptom of dysphagia is already present. Data obtained indicate the need for an assessment of the nutritional status already at the diagnosis of HNC and especially before surgery, chemotherapy, and radiotherapy; this is essential to properly feed the patient to avoid the onset of malnutrition and related complications and above all to ensure a better clinical recovery after surgery. Furthermore, constant monitoring of nutritional status and evaluation of both anthropometric and biochemical parameters are necessary to identify the best type of nutrition for the patient. The evolution of the disease, the surgical interventions or the adverse effects of chemo-radiotherapy must be considered to improve the survival and quality of life of these patients. In conclusion, even if this observational study has limitations such as the small number of patients evaluated, it indicates the need for close collaboration between different professional figures, such as the oncologist, the maxillofacial surgeon, and the nutritionist for a better management of the patient with head and neck cancer.

**Table 1:** Demographic and anthropometric characteristics of the population divided according to the diagnosis of dysphagia

Variables	A Group (without dysphagia; n = 31)	B Group (with dysphagia; n = 29)	*p-*value
Age (years)	70 ± 15	67 ± 8	0.39
Gender, male (%)	52	79	0.032
Weight (kg)	65.1 ± 11	60.8 ± 13	0.20
BMI (kg/m^2^)	25.3 ± 5	20.3 ± 4	< 0.001
Lost weight vs usual weight (%)	8.1 ± 7	16.4 ± 9	< 0.001
Arm circumference (cm)	28.1 ± 4	24.3 ± 3	0.002
Triceps fold (cm)	1.3 ± 0.7	0.8 ± 0.5	0.003
Handgrip (kg)	20.6 ± 9	20.6 ± 10	0.99
PG-SGA	9.8 ± 7	13.2 ± 6	0.07
Phase angle (°)	4.40 ± 1.2	4.45 ± 0.8	0.89
TBW (%)	55.4 ± 8	62.3 ± 8	0.040
FM (%)	25.7 ± 10	16.4 ± 9	0.019
SMI (kg/m^2^)	8.4 ± 1	8.7 ± 2	0.73
SMM (kg)	20.2 ± 3	23.3 ± 7	0.11
ASMM (kg)	15 ± 2	16 ± 4	0.20
basal metabolic rate (kcal)	1282 ± 163	1330 ± 147	0.38

**Table 2:** Characteristics of the blood chemistry parameters of the population divided according to the diagnosis of dysphagia

Variables	A Group (without dysphagia; n = 31)	B Group (with dysphagia; n = 29)	*p-*value
Glucose (mg/dL)	106 ± 28	100 ± 23	0.42
Creatinine (mg/dL)	0.85 ± 0.3	0.87 ± 0.4	0.89
Total cholesterol (mg/dL)	179 ± 42	172 ± 47	0.60
HDL-Col (mg/dL)	50 ± 15	44 ± 16	0.25
Triglycerides (mg/dL)	140 ± 77	127 ± 54	0.52
Calcium (mg/dL)	9.3 ± 0.5	9.1 ± 1	0.54
Phosphorus (mg/dL)	3.5 ± 0.5	3.4 ± 1	0.94
Sodium (mmol/L)	139 ± 4	139 ± 3	0.88
Iron (mcg/L)	62 ± 22	56 ± 30	0.53
Total proteins (g/dL)	6.9 ± 0.6	6.0 ± 0.7	0.92
Albumin (g/dL)	4.1 ± 0.5	3.6 ± 0.6	0.004
Red blood cells (× 10^3^/uL)	6.3 ± 1.9	8.3 ± 4.4	0.049
White blood cells (× 10^6^/uL)	4.6 ± 0.6	3.9 ± 0.6	< 0.001
Haemoglobin (g/dL)	13 ± 1.4	11 ± 1.6	0.001

## Data Availability

The data presented in this study are available on request from the corresponding author.
